# First trimester determination of fetal gender by ultrasonographic measurement of anogenital distance: A cross-sectional study

**DOI:** 10.18502/ijrm.v17i1.3820

**Published:** 2019-03-07

**Authors:** Nazila Najdi, Fatemeh Safi, Shahrzad Hashemi-Dizaji, Ghazal Sahraian, Yahya Jand

**Affiliations:** ^1^Department of Gynecology and Obstetrics, School of Medicine, Arak University of Medical Sciences, Arak, Iran.; ^2^Department of Gynecology and Obstetrics, Shahid Akbarabadi Hospital, Faculty of Medicine, Iran University of Medical Sciences, Tehran, Iran.; ^3^Departement of Pharmacology, School of Medicine, Tehran University of Medical Science, Tehran, Iran.

**Keywords:** *Sonography*, * Gender*, * Female*, * Male*, * Pregnancy*, * First trimester.*

## Abstract

**Background:**

In some patients with a family history of the gender-linked disease, determination of the fetal gender in the first trimester of pregnancy is of importance. In X-linked recessive inherited diseases, only the male embryos are involved, while in some conditions, such as congenital adrenal hyperplasia, female embryos are affected; hence early determination of fetal gender is important.

**Objective:**

The aim of the current study was to predict the gender of the fetus based on the accurate measurement of the fetal anogenital distance (AGD) by ultrasound in the first trimester.

**Materials and Methods:**

To determine the AGD and crown-rump length in this cross-sectional study, 316 women with singleton pregnancies were exposed to ultrasonography. The results were then compared with definitive gender of the embryos after birth.

**Results:**

The best cut-off for 11 wk to 11 wk, 6 days of pregnancy was 4.5 mm, for 12 wk to 12 wk, 6 days was 4.9 mm, and for 13 wk to 13 wk, 6 days was 4.8 mm.

**Conclusion:**

AGD is helpful as an ultrasonographic marker that can determine fetal gender in the first trimester, especially after 12 wks.

## 1. Introduction

For patients with a family history of gender-linked diseases, determination of the fetal gender in the first trimester of pregnancy is very important. In X-linked recessive inherited diseases, only male fetuses are involved, and in adrenal congenital hyperplasia, only female fetuses are involved. It is clear that early detection of fetal gender is of importance. The most definitive method of determining the gender of an embryo is the biopsy of the chorionic villus under ultrasound, but this is an invasive procedure with a 0.5% to 1.0% chance of fetal loss (1, 2). Another method of determining gender is to analyze the cell-free fetal DNA in the mother's blood. This is considered to be a non-invasive procedure, but is costly and is not available in all locations (3).

The simplest non-invasive technique to determine the fetal gender is ultrasonography in the second trimester and the use of the morphological criteria (penis and scrotum for males and labia major and minor in females). In the absence of gender abnormalities, this easy and available technique is 100% accurate after 20 wk of pregnancy (4–6). Before the second trimester of pregnancy, the accuracy of fetal gender determination decreases due to biological factors such as a small size and insufficient difference between genitalia or because of technical factors such as the difficulty of correct determination of the midsagittal plane (7, 8).

In the late 1990s, a new technique was developed for the determination of fetal gender in the first trimester. This involved measurement of the genital angle of the tubercle to a horizontal line on the lumbosacral skin in the midsagittal plane of the fetus (9, 10). After 13 wk of pregnancy, this procedure has a sensitivity of 100% for determining gender, but its sensitivity between 11 and 12 wk is low (4, 9, 11, 12).

Gender morphogenesis is a dynamic and hormone-dependent phenomenon that occurs after 6 wk of pregnancy. The anogenital distance (AGD), the distance between the tail-like end of the fetus and the genital base of the tubercle, is dependent on testosterone and, thus, on the gender (13). The AGD of males is larger than of females (14, 15). In animal models, this distance represents fetal exposure to androgens during masculinization (16, 17). In humans, AGD in male neonates is more than twice of that in females. This difference is significant until the age of 24–30 months, after which it decreases up to adulthood (14, 15, 18).

The necessity for early diagnosis of fetal gender as related to gender-dependent diseases was the motive for the present study to evaluate the accuracy of AGD ultrasonographic measurement at 11 wk to 13 wk, 6 days of pregnancy.

## 2. Materials and Methods

A total of 316 pregnant women referred to Taleghani Hospital, Arak, Iran, from October 1, 2016 to September 1, 2017 offered to participate in this cross-sectional study.

**Figure 1:**
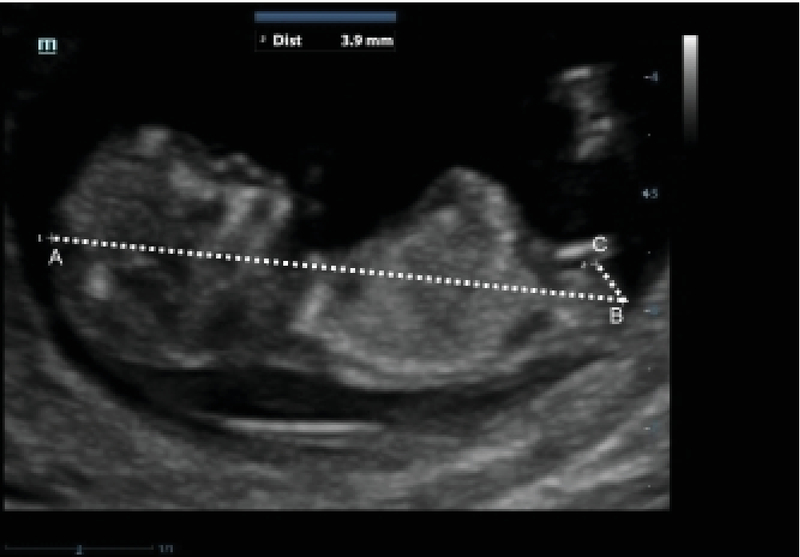
Measurement of CRL (line AB) and AGD (line BC), (magnification 1500×1125).

Our inclusion criteria were women aged 18–35 years old with a singleton pregnancy in 11 wk to 13 wk + 6 days. The exclusion criterion was patient failure to provide consent. The demographic data of the mothers were collected on an information form. All mothers submitted to ultrasound by an experienced sonologist during which the fetal AGD was examined in a neutral position (neither hyperflex nor hyperexten) on the midsagittal plane. The image was used to measure the crown-rump length (CRL) (13) to determine the exact age of the pregnancy. The AGD then was measured from the fetus caudal to the inferior end of the genital appendage (Figure 1). Specifications of ultrasound instrument that was used in this study contain: Probe Convex c7-3, zooming 500%.

To assess the participants, they were divided into three groups by the age of pregnancy:

• Group 1: women with pregnancy age 11 wk to 11 wk, 6 days (*n* = 87),
• Group 2: women with pregnancy age 12 wk to 12 wk, 6 days (*n* = 180), and
• Group 3: women with pregnancy age 13 wk to 13 wk, 6 days (*n* = 48).

The ROC curves were used to define a cut-off for AGD in each group. The baby's gender of all the participants was recorded.

### Ethical consideration

The procedure and goals of the project were explained to all participants and then they were asked to sign the informed consent forms. The protocol of this study was confirmed and approved by the Ethics Committee of the ARAK University of Medical Sciences (IR.ARAKMU.REC.1395.348).

### Statistical analysis

Statistical analysis data was entered into SPSS version 15 (USA). ROC analysis was used to calculate the predictive value of some variables. These values were considered to be statistically significant at p < 0.05.

## 3. Results

The follow-up process was completed for all 316 pregnant women under the study. A total of 87 fetuses were studied in group 1. Out of this number, 60 embryos were female with an average AGD of 4.01 ± 0.81 mm and 27 fetuses were male with an average AGD of 4.68 ± 0.74 mm (p = 0.0019). The average CRL of the female fetuses was 46.88 ± 2.65 mm and of the male fetuses was 49.68 ± 2.67 mm; thus, there was no significant difference between them. Group 2 included fetuses of 12 wk to 12 wk, 6 days of age. A total of 180 fetuses were studied. From this number, 102 fetuses were female with an average AGD of 4.25 ± 0.57 mm and 78 were male with an average AGD of 4.67 ± 0.59 mm (p < 0.00001). No significant difference was observed between the average CRL of male and female fetuses.

Group 3 included the fetuses of 13 wk to 13 wk, 6 days of age. These totaled to 48 fetuses. Of these, 23 fetuses were female and 27 were male. The average AGD of the females was 4.65 ± 0.71 mm and of the boys was 6.06 ± 0.85 mm (p < 0.0001). There was no significant difference in the average CRL between the male and female fetuses of this group.

**Figure 2:**
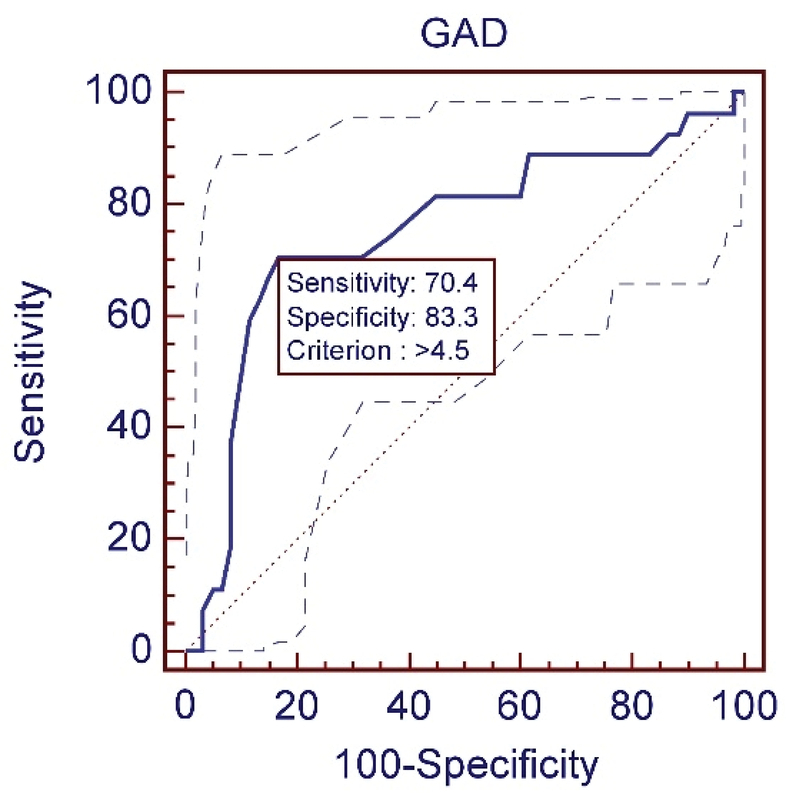
ROC curve of the anogenital distance; measured in fetuses with the pregnancy age of 11 w to 11w + 6d.

**Figure 3:**
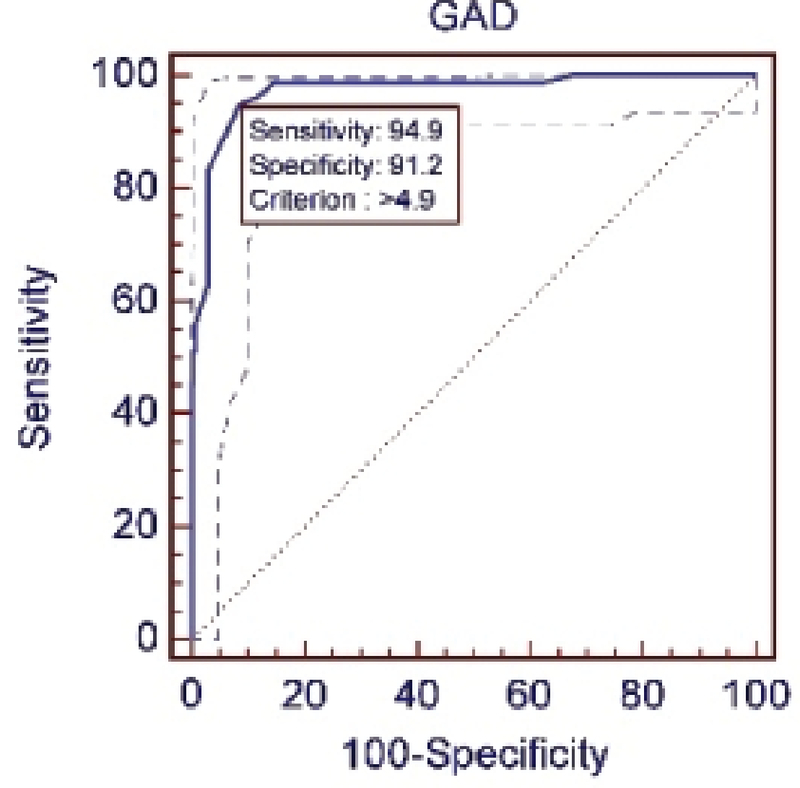
ROC curve of the anogenital distance; measured in fetuses with the pregnancy age of 12 w to 12w + 6d.

**Figure 4:**
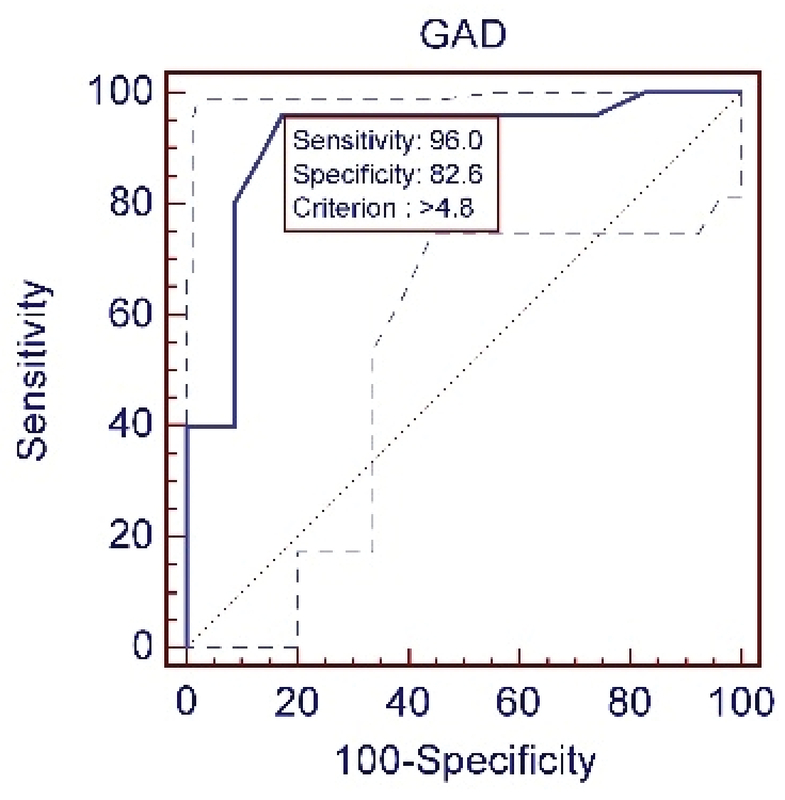
ROC curve of the anogenital distance; measured in fetuses with the pregnancy age of 13 w to 13w + 6d.

**Figure 5:**
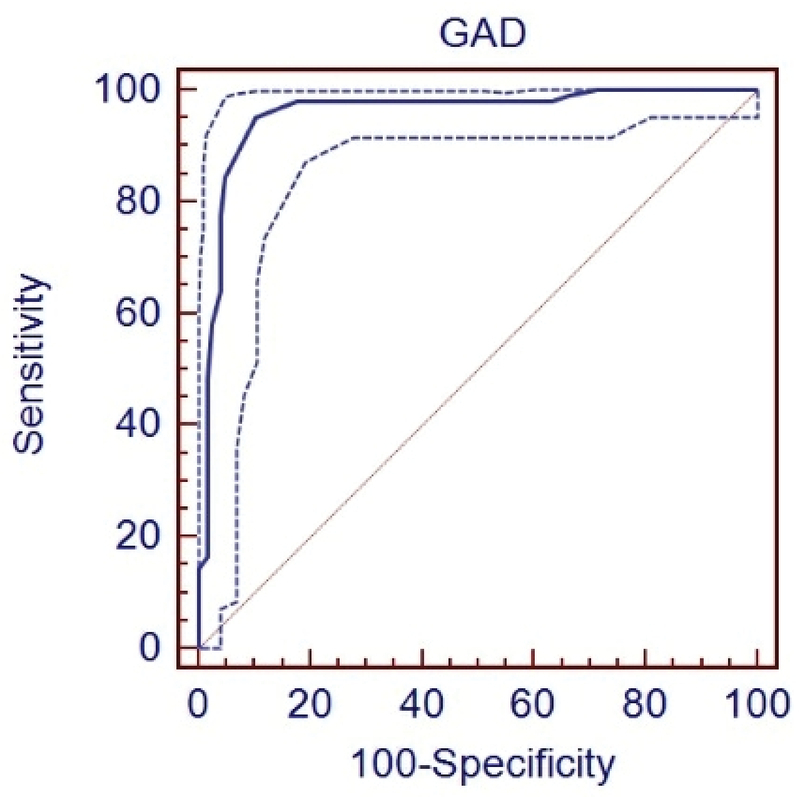
ROC curve of the anogenital distance; measured in fetuses with the pregnancy age of 12 w to 13w + 6d.

To remove the effect of age on AGD for each group, the best cut-off was separately calculated for predicting gender (Figures 2–4). Characteristics and sensitivity were determined in all three groups (Table I). The areas under the curve were compared every 3 wks. No significant difference was observed between groups 2 and 3 (p = 0.10). A significant difference was observed between groups 2 and 3 and group 1 (p = 0.0003); thus, the samples in groups 2 and 3 were combined into one group. The best cut-off was determined to be 4.9 mm (Figure 5). The sensitivity and specificity were calculated, respectively, as 95% and 89.6% (Table II).

**Table 1:** Characteristics and sensitivity were determined in all three groups.


**GA**	**Criterion**	**Sensitivity**	**92%CI**	**Specificity**	**92%CI**	**+LR**	**92%CI**	**–LR**	**92%CI**	**+PV**	**92%CI**	**–PV**	**92%CI**	**AUC**
11W	> 4.5mm	70.37	49.8–86.2	83.33	71.5–91.7	4.22	3.2–5	0.36	0.2 = 0.8	65.5	45.7–82.0	86.20	74.6–93.8	0.748
12W	> 4.9mm	94.87	91.1	91.18	83.9–95.9	10.75	9.9–11	0.05	0.02–0.2	89.2	80.4–94.9	95.90	89.8–98.8	0.972
13W	> 4.8mm	96.00	82.61	82.61	61.2–94.9	5.52	4.5–6	0.04	0.006–0.4	85.7	67.3–95.9	95.00	75.1–99.2	0.913
Note: GA: Gestational age; CI: Confidence interval; +LR: Positive likelihood ratio; –LR: Negative likelihood ratio; +PV: Positive predictive value; –PV: Negative predictive value; and AUC: Area under the curve.														

**Table 2:** Characteristics and sensitivity were determined in fetuses with pregnancy age of 12w to 13w + 6d.


**Criterion**	**Sensitivity**	**92%CI**	**Specificity**	**92%CI**	**+LR**	**92%CI**	**–LR**	**92%cI**	**+PV**	**92%CI**	**–PV**	**92%CI**
> 4.9mm	95.15	89.0–98.4	89.6	82.9–94.3	9.15	8.5–9.9	0.054	0.02–0.1	88.3	80.8–93.6	95.70	90.3–98.6

## 4. Discussion

The results of this cross-sectional study suggest that ultrasonographic measurement of the AGD of embryos in the first trimester of pregnancy is a novel and accurate means to determine embryo gender. The AGD in the male embryos was significantly greater than that in the female embryos. The best results were obtained in pregnancies of over 12 wk of age at a cut-off of 4.9 mm. On this basis, the gender of 88% male embryos and 95% female embryos was diagnosed correctly. The measurement of AGD at 11 wk of pregnancy contained the weakest result.

Salazar-Martines and co-workers measured AGD in male and female neonates. They examined 87 neonates (42 girls and 45 boys) resulting in term pregnancy (38 ≥ wk). In all the neonates, AGD was measured. The results showed that the AGD of male neonates (average 22 mm) was twice as that of the female neonates (average 11 mm) (14).

Kutlu studied AGD in Turkish neonates. The AGD of 300 neonates from two studies were measured. The AGD of the males was 41.8 ± 4.9 mm and of the females was 35.04 ± 3.34 mm that showed a significant difference between genders (19). Few studies have been done on the difference of AGD between male and female embryos. Arfi and co-workers determined the gender of fetuses in the first trimester of pregnancy by measuring AGD. The genders of the fetuses of 310 women with singleton pregnancies were evaluated at 11 wk to 13 wk, 6 days of pregnancy. By measuring AGD, the researchers attempted to determine a cut-off for accurate determination of the gender of the fetus. The ROC curve was used to calculate this cut-off as being 4.8 mm. The chance of predicting a male embryo at an AGD of ≥ 4.8 mm was 92.3% and at AGD ≤ 4.8 mm, the chance of predicting a female embryo was 89.8% (13).

In the current study, to increase the precision of the results, embryos studied in the first trimester of pregnancy were divided into three groups to determine the best week of pregnancy for determining fetal gender based on AGD. The weakest results were for 11 wk to 11 wk, 6 days, at a cut-off of 4.5 mm. The likelihood of the embryo being male at AGD < 4.5 mm was 65%. The likelihood of an embryo being female at AGD ≥ 4.5 mm was 81%. The best outcome was at more than 12 wk with a cut-off of 4.9 mm. In this case, the likelihood of prediction of a male embryo at AGD > 4.5 mm was 89.2% and of predicting a female embryo at AGD ≥ 4.9 mm was 95.9%.

None of the previous studies for the determination of the gender of a fetus by AGD had been done in the first trimester. The current prospective study supports the measurement of fetal AGD as a new ultrasonographic sign for early determination of the gender of the fetus. It appears that this method is an accurate tool, especially after 12 wk of pregnancy. More studies are needed to verify the results.

##  Conflict of Interest

The authors declare that they have no conflict of interest in this study.
